# Investigation of the effect of Low‐Level Laser Therapy on arm lymphedema in breast cancer patients: A noninvasive treatment for an intractable morbidity

**DOI:** 10.1002/hsr2.1261

**Published:** 2023-05-17

**Authors:** Farshid Farhan, Mahmood Samei, Alireza Abdshah, Ali Kazemian, Shahriar Shahriarian, Farnaz Amouzegar‐Hashemi, Mostafa Farzin, Reza Ghalehtaki, Fatemeh Jafari, Francesco Cuccia

**Affiliations:** ^1^ Radiation Oncology Research Center (RORC), Cancer Institute Tehran University of Medical Sciences Tehran Iran; ^2^ School of Medicine Tehran University of Medical Sciences Tehran Iran; ^3^ Department of Public Health Sciences Division of Biostatistics, University of Miami Miller School of Medicine Miami Florida USA; ^4^ Department of Radiology, Imam Khomeini Hospital Complex Tehran University of Medical Sciences Tehran Iran; ^5^ Brain and spinal cord injury research center, Neuroscience Institute Tehran University of Medical Sciences Tehran Iran; ^6^ Department of Radiation Oncology, Cancer Institute, Imam Khomeini Hospital Complex Tehran University of Medical Sciences Tehran Iran; ^7^ Advanced Radiation Oncology Department Sacro Cuore Don Calabria Hospital Negrar Veneto Italy

**Keywords:** breast cancer, low‐level laser, lymphedema

## Abstract

**Purpose:**

This article aims to study the effect of Low‐Level Laser Therapy (LLLT) on arm lymphedema in patients who have breast cancer.

**Methods and Materials:**

Twenty‐three patients were selected in a nonrandomized phase‐2 clinical trial. After measuring the circumference of the affected and unaffected limbs at 6‐points, the volume of the limbs, the degree of mental symptoms on visual analog scale by the patient upon entering the study, and performing an ultrasound on the patient's axilla to locate the fibrotic areas, a low‐level laser device at a therapeutic dose of 2 J/cm^2^ was used to treat the patients three times a week for 4 weeks, and after an 8‐week gap, for another similar period. Measurement of circumference and volume of affected and unaffected limbs and mental symptoms were carried out at the end of the 4th week, the beginning of the 12th week, and the end of the 16th week, and the obtained results were compared with those before the treatment.

**Results:**

We noted that the average reductions in the circumference and volume of the affected limb, as compared with the unaffected limb, were about 16% and 21.7%, respectively, and improvement in the patient's mental symptoms was about 32%. Another notable observation was the great enthusiasm of most patients to continue their treatment, particularly from the second cycle onward.

**Conclusions:**

LLLT can, at least in association with current standard methods, be used for arm lymphedema to introduce further reductions in pain and volume.

## 1 INTRODUCTION

Breast cancer is one of the most common malignancies afflicting women in the world and Iran. The annual incidence rate in Iran is approximately 31.6 per 100,000.^1^, ^2^ The standard treatment is surgery (either mastectomy or breast‐conserving surgery) with or without chemotherapy or radiotherapy. Thanks to the effectiveness of today's therapies (surgery, chemotherapy, and radiotherapy), the life span of cancer patients has increased dramatically; however, many of the patients will, unfortunately, need to live the rest of their lives with the potential complications resulting from the cancer treatment, which can adversely affect their quality of life. Arm lymphedema is one of the most common complications in breast cancer treatment that could seriously disrupt the affected limb's functioning and cause psychological morbidity. In the cases where dissection and radiotherapy of both axillae are performed, the risk of arm lymphedema is significantly increased.^1^, ^3^, ^4^

Lymphedema results from dysfunction of the lymphatic system, which removes the excess extravascular and interstitial fluid that has been excreted from the capillary system into the tissues. The excessive accumulation of extracellular liquids and proteins causes further pressure on the blood vessels and nerves, reduces skin flexibility, and adversely affects the limb's performance. Pain, paresthesia, and reduction of free joint movement would bring about dissatisfaction with life. Therefore, finding a suitable, effective, inexpensive, and even more importantly accessible therapy is a priority for health systems worldwide.

Different methods have been utilized for managing lymphedema, but no definitive treatment has so far been identified for this complication. Recently, Low‐Level Laser Therapy (LLT) has introduced a new and a promising era in the treatment of this complication Historically, treatments such as massaging, using compression devices, and bandaging of the limb, along with physiotherapy and medications have been investigated before, and many articles have been published about them. Using medication to reduce fibrosis was considered after studying the pathophysiology of postsurgery and postradiotherapy arm lymphedema. These phenomena result from damage to lymphatic pathways which causes post‐treatment fibrosis. Some investigators like Delanian et al. tried to eliminate the fibrosis by intramuscular injection of liposomal Cu/Zn superoxide dismutase.^5^ Lately, reports indicating reduction of fibrosis due to LLLT, have given rise to the idea that a fibrotic lymphatic pathway could be repaired with this kind of treatment as well.

According to a recent systematic review by Mahmood et al. LLT showed promising results in decreasing arm circumflex, but did not reduce shoulder mobility or pain significantly.^6^

## 2 MATERIALS AND METHODS

Twenty‐three breast cancer patients with previous surgery (mastectomy or breast‐conserving surgery) and radiotherapy were selected. The minimum difference in displaced water volume between the affected and unaffected limbs was 200 mL, or the minimum of the average difference between affected and unaffected limbs' circumference, measured at six different locations of the limbs with a measuring tape was 2 cm or 10% (the difference percentage is calculate by dividing the difference between the volume or circumference of the affected and unaffected limbs by the volume or circumference of the unaffected limb, multiplied by 100). All the patients were briefed about the treatment procedure and consequently submitted their written informed consent and agreed to undergo LLLT for an overall period of 2 months with an 8‐week interval in between. As it was deemed unethical to deny treatment to a control group who would come and go to and from the medical center in hopes of being treated, and as arm lymphedema is a persistent and progressive complication that does not go away by itself, it was decided, upon consultation with relevant medical experts, that there should be no control group in this study, and that patients should be compared to themselves (arms compared to each other) as “pre‐treatment” and “post‐treatment” cases.

The criteria for admission to the study included the following: persistence of lymphedema following mastectomy or breast conservative surgery for at least 3 months, and the existence of either a minimum volume difference of 200 mL or an average difference in arm circumference of 2 cm or 10% between healthy and affected limbs.

Exclusion criteria consisted of metastatic or recurrent disease, established vein thrombosis, history of severe arm trauma, chronic inflammatory disease, congenital lymphedema, recent infection, and drugs that affect body fluid balance, pregnancy, and photophobia.

### 2.1 Study protocol

**Figure 1:**
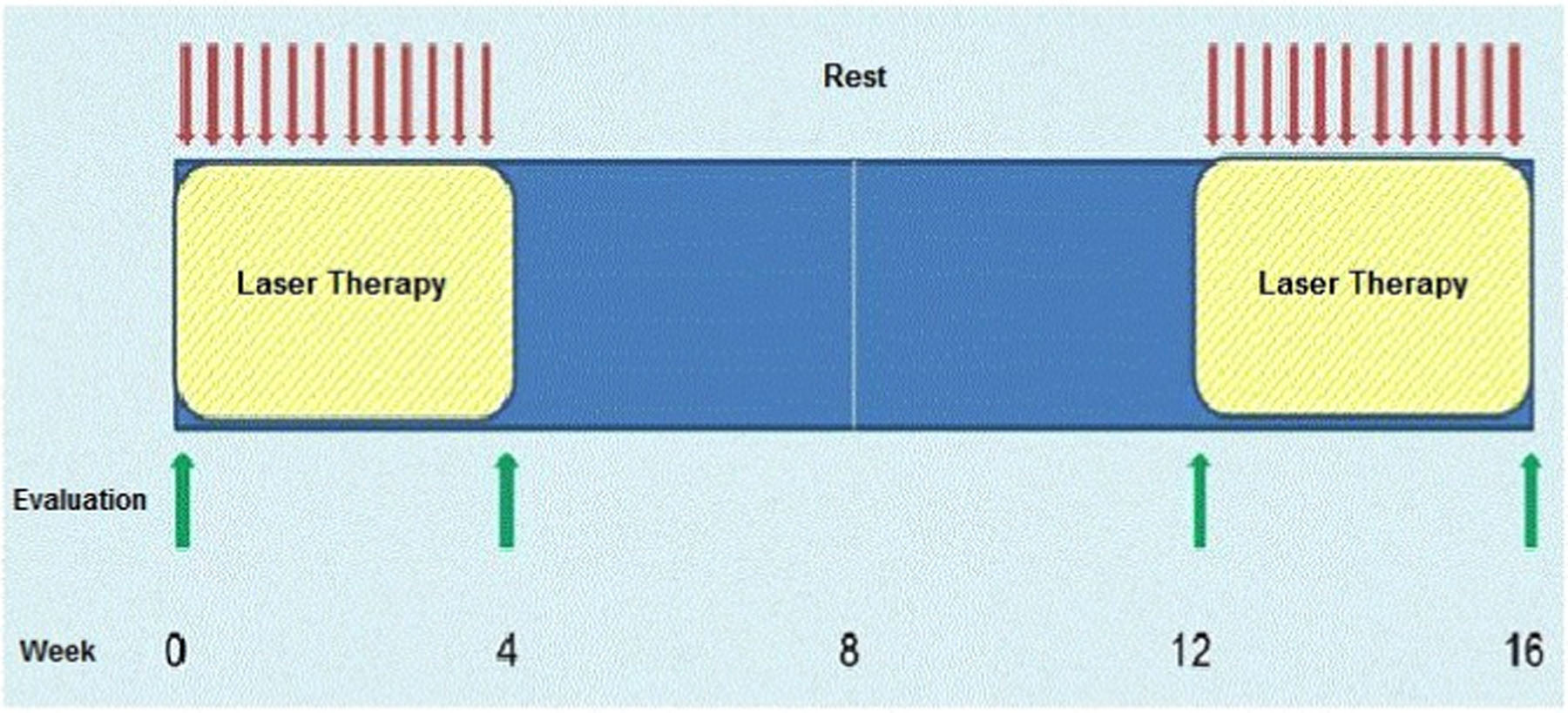
Treatment protocol.

All patients considered eligible for the study were, before the study's onset and for exact determination of fibrotic areas, examined by a skillful radiologist through ultrasound. The mentioned areas were marked in ink so that the treatment area could be easily distinguished. The treatment protocol is shown in Figure 1.

Patients were subjected to measurement at Week 0, end of Week 4, the start of Week 12, and end of Week 16, and their limb circumferences measured with measuring tape at 6‐points: roots of fingers, the wrist, 5 cm above the wrist, the elbow, 5 cm above and 5 cm below the elbow and the average differences between the afflicted and healthy limbs were recorded. Also, the difference in the limbs' volume was determined and recorded through the water volume displacement method. This was done by the trained, skillful personnel of the Physiotherapy Ward. The patients would undergo treatment three times a week for 4 weeks with a low‐level laser device of Spotting Pen type (Heltschel Company), with a radiation dose of 2 J/cm^2^, an output of 100 mWatt, and a wavelength of 658 nanometers. The Food and Drug Administration has not declared this laser therapy device for the treatment of postmastectomy lymphedema, and it has been used off‐label. After an 8‐week rest period, the treatment was resumed for another 4 weeks, coming up to a total of 24 sessions. To ensure our ability to correctly localize the treated area for the next sessions, sheets of radiology films were prepared in which holes were made with a spacing distance of 2 cm and diameters equal to the laser apparatus therapeutic head. Each treatment point would be irradiated for 80 s. The treatment time was calculated from the following formula:

$$\begin{array}{lll} \text{Treatment}\;\text{Time}\;\text{in}\;\text{Seconds}\; & = & \text{Cross}\;\text{Section}\;\text{Area}\;{(\text{cm}}^{2})\\ & \times & \text{Radiation}\;\text{Dose}\;{(J/\text{cm}}^{2})/\text{Power}\;(W). \end{array}$$

The patients and the therapist used protective goggles during all therapeutic sessions. The area of treatment was determined by the radiologist and then divided into 16−24 points for treatment.

### 2.2 Patient evaluation

Evaluation of the patients was conducted at the end of the 4th week, the start of the 12th week, and end of the 16th week, through measuring the changes produced in the volume and circumference of the affected limbs relative to the healthy side, as well as the mental symptoms (pain, stiffness, paresthesia, heaviness, and organ infirmity), and then comparing them to the corresponding values measured on the first day. The average changes in the circumference and volume of the affected limbs were calculated, in percentage, from the following formula:

[(VCAO−VCUO)/(VCUO)] × 100

Where:

VCUO: Volume or circumference of the unaffected organ

VCAO: Volume or circumference of the affected organ

The patients' subjective symptoms were recorded on the visual analog scale (length = 20 cm) at the onset of the treatment and the end of the 4th, 11th, and 16th weeks. Their mean (average) values expressed as percentages, that is, symptoms such as pain, stiffness, paresthesia, heaviness, and limb weakness, were tested separately, and their average values were determined as a single percentage to represent subjective changes.

This was a phase‐2 clinical trial; therefore, no randomizations were performed. Sample size was calculated using a typical Gehan design. Considering the minimum efficacy of 20% in patients (*p* = 0.2) and alpha=10%, using the Gehan design, we would need 11 patients (10.3 rounded to 11) in the first step, and a maximum of 11 other patients if we observe effect in the first set of patients. The patients were treated in a single institution and all the analyses were conducted using the SPSS Software, Version 11.5, and the *p* Value under 0.05 was considered the statistically significant value. Variations in quantitative values and the difference between the basic values and the values obtained during the study were evaluated through the Wilcoxon Signed Rank Test and the Paired‐*T* Test. The Mann−Whitney test was used to investigate changes in the secondary variables such as weight, age, afflicted side of patient's body, body mass index, lymphedema severity, number of removed lymphatic nodes, and type of surgery radiotherapy, and background diseases.

This trial is approved by the ethics committee of Tehran Medical School.

## 3 RESULTS

From February 2011 through March 2012, that is, for 14 months, 23 patients were chosen for the study. During the study, one patient developed distant metastasis at the end of the 4th week and was excluded, and two patients discontinued treatment due to personal reasons. The remaining 20 patients managed to complete the two laser treatment cycles. The average age of patients was 49 years, and the average severity of lymphedema was 49% (volume or circumference increase of the afflicted arm relative to the healthy side).

The basic and demographic information about patients is given in Table 1, and the results obtained during treatment are recorded in Table 2. As seen in these tables, the difference in terms of average volume and circumference between the afflicted and the healthy limbs at the onset of the treatment was statistically significant with a P‐Value of less than 0.05. The decrease in volume difference between the afflicted and the healthy limbs was more than 200 mL in 59% of the patients, and the decrease in the circumference difference between the afflicted and the healthy limbs was more than 0.5 cm in 53% of the patients. If we compare the average volume and circumference of the afflicted limb before and after treatment, an average volume reduction of 200 mL, that is, 21.7%, and an average arm circumference reduction of 0.5 cm, that is, about 16%, can be observed at the end of the 16th week compared to the first day of treatment, which is statistically significant with a *p* Value of less than 0.05. However, if the calculations are based on the volume and circumference reductions relative to those of healthy limbs at the end of the 16th week, then the corresponding results would be 9% and 3.5%, which, based on a *p* Value greater than 0.05, would not be statistically significant.

**Table 1:** Demographic and baseline characteristics of patients.

	Mean (SD)	Min	Max
Age	49 (7.3)	27	76
Lymphedema severity	45% (11.8%)	10%	110%
Body mass index	27 (3.3)	19.5	43
Number of removed lymph nodes	9 (2.6)	2	25
Months since radiotherapy	25 (5.8)	3	83
Months since surgery	38 (7.2)	18	97
Type of surgery	Mastectomy (9, 45%)	Breast‐conserving surgery (11, 55%)	
The affliction of the dominant hand	Dominant (8, 40%)	Nondominant (12, 60%)	
Type of radiotherapy	Tangential (6, 30%)	Tangential and supraclavicular (14, 70%)	

**Table 2:** Results of the treatment in patients.

Parameter	Baseline	Week 4	Week 12	Week 12	*p* Value within group	Week 12 to baseline change rate
ΔV	920	810	850	720	0.013	200
	48%	40%	43%	39%		21.73%
ΔC	3.15	2.8	2.85	2.65	0.039	0.5
						16%
	23%	20%	20%	19.5%		
Change in psychological symptoms	0	20%	27%	32%	0.026	32%
Pain reduction	0	30%	37%	45%	0.001	45%
Stiffness reduction	0	45%	52%	55%	0.000	55%
Paresthesia reduction	0	10%	15%	20%	0.023	20%
Heaviness reduction	0	15%	22%	25%	0.008	25%
Limb weakness reduction	0	0	10%	15%	0.039	15%

Abbreviations: ∆V, Average difference in volume between the affected and healthy arms in mL and %; ∆C, Average difference in circumference between the affected and healthy arms in cm and %.

A closer look at lower rows in Table 2 shows that changes in subjective symptoms are much more visible, and on average, patients have reported a 32% improvement in these symptoms. Focusing on the mental symptoms individually, suggests that treatment has had a greater effect on pain and stiffness than on paresthesia, heaviness, and limb weakness. Moreover, except for two minor nausea and feeling of weakness, no other complications were reported by the patients.

**Figure 2:**
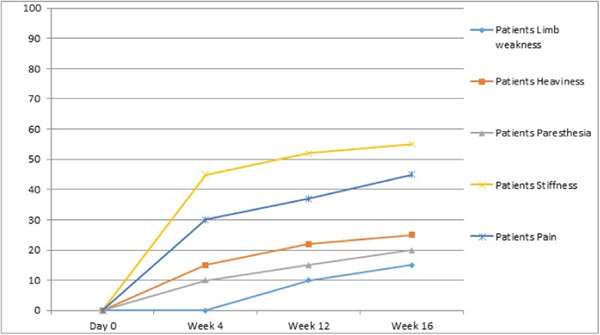
Variation trend in mental symptoms.

**Figure 3:**
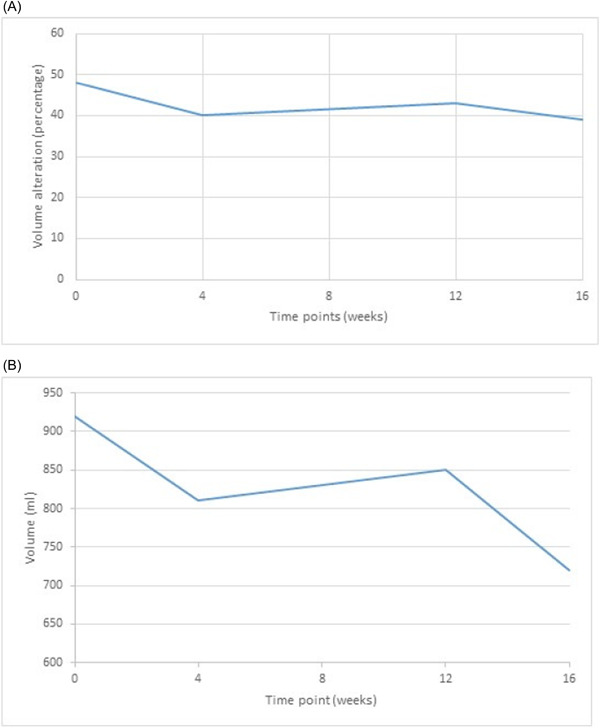
(A, B) Variation trends in quantity (A) and percentage (B) of the afflicted arm volume.

**Figure 4:**
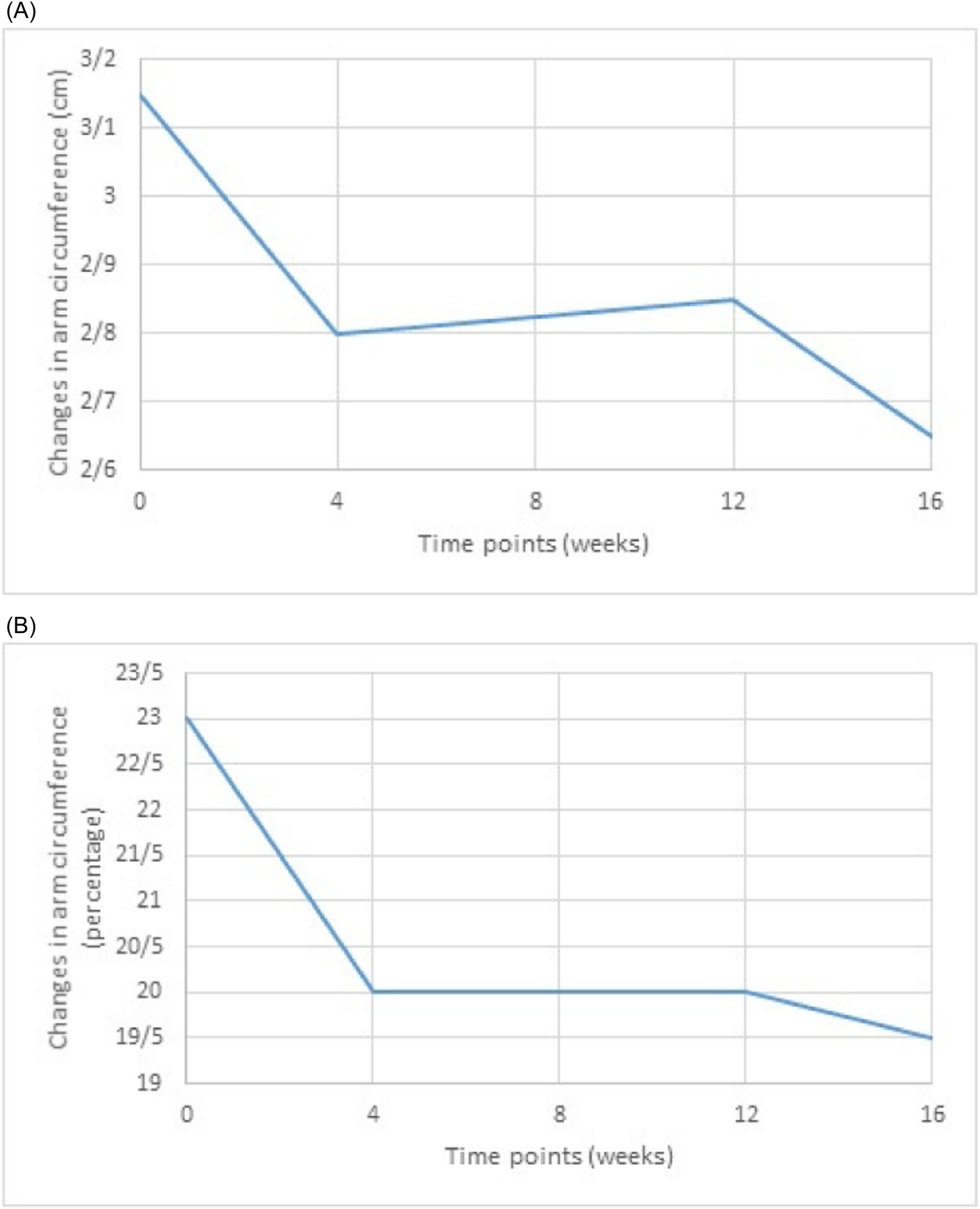
(A, B) Variation trends in quantity (A) and percentage (B) of the afflicted arm circumference.

We can observe in Table 3, that the results of LLLT in patients with fewer removed lymph nodes and lesser degree of lymphedema, is more significant and the reduction in variations recorded for volume and circumference difference between afflicted and healthy limbs is greater. This observation might be due to the existence of high interstitial pressure in severely damaged cases, which does not allow any appreciable reduction in volume and arm circumference despite the reduction in edema and stiffness. However, as we have not used skin tonometry in designing our study, we are unable to support this claim with experimental evidence. The same problem exists in patients with a cancer treatment history extending further into the past, that is, those patients with longer duration of their symptoms, who had probably suffered more severe lymphedema, responded less favorably to the treatment. Figures 2, 3, and 4 show the variations of mental symptoms and the volume and circumference of the limbs.

**Table 3:** Results in terms of individual secondary parameters, comparing different categories changes in volume and circumference.

Results	Average	∆V and ∆C In patients above average	*p* Value	∆V and ∆C In patients Below Average	*p* Value
Age	49	21.9%−15.9%	0.127	20.4%−16.2%	0.233
Body mass index	27	21.5%−16.3%	0.251	22.1%−15.9%	0.301
Number of resected lymph nodes	9	17.8%−13.9%	0.747	25.4%−19.2%	**0.030**
Number of months passed since the last treatment	25	12.9%−9.4%	0.430	32.1%−23.5%	**0.001**
Lymphedema severity	45	16.7%−11.9%	0.119	29.6%−19.8%	**0.007**
		∆V−∆C Dominant Hand		∆V−∆C nondominant hand	
Dominant hand	‐	22%−16.1%	0.259	21.3%−16.5%	**0.046**
		Tangential ∆V−∆C		Tangential and Supraclavicular ∆V−∆C	
Type of radiotherapy	‐	21.8%−16%	0.711	21.2%−15.9%	0.167
		Mastectomy ∆V−∆C		Breast‐Conserving ∆V−∆C	
Type of surgery	‐	21.2%−15.5%	0.283	22.3%−16.5%	0.451

Abbreviations: ∆V, average change in arm volume; ∆C, average change in arm circumference.

## 4 DISCUSSION

Our findings reveal improvements in arm volume, arm circumference and subjective symptoms by 21.7%, 16%, and 32% with PBMT, respectively. This could suggest the effectiveness of photobiomodulation in treating lymphedema. Some preclinical studies have suggested the underlying mechanism of laser effectiveness in lymphedema. Kiyoizumi et al.^7^ in an in‐vitro experiment reported that the GaAlAs laser stimulates prostacyclin (PGI2) synthesis, which itself has a strong vasodilator effect and prevents platelets aggregation and thereby reduces edema. Lievens et al.^8^ in another study used GaAs/HeNe laser and showed that the lymphatic vessels regenerated faster and more steadily into their original shape, and not in the shape of small vessels, after laser therapy.

Several clinical studies have reported results in concordance to our findings. In the study conducted by Carati et al.^9^ 64 patients, divided in two groups, were tested. At 1‐ and 3‐months follow‐up sessions after two cycles of laser treatment, 31% of treated cases showed at least 200 mL volume reduction as compared with 4% of the placebo group. In addition, 52% of the patients in case group showed reduced skin tightness as compared with 24% in the placebo group. The average volume reductions in the treatment and placebo groups were 89.7 and 32.1 mL respectively, and their difference amounted to 57.6 mL. As in our study, total average volume reductions in patients were 200 mL. A study by Mohammad Taher et al.^10^ showed that laser therapy led to limb volume reduction in 93% of patients and recovery of handgrip strength. In that study, the average circumference reductions in the affected limbs in the treatment and control groups were 31% and 23%, respectively (compared to healthy limbs), with a difference equal to 8%. In our study, reduction in circumference was 16%. Some reports have stated much higher rates for circumference reduction after laser therapy. For instance, Dirican et al.^11^ have conducted two treatment cycles with 904‐nanometer low‐level Lithium laser on 34 patients and have ended up with reduced rates of 54 and 73 in limb circumference after first and second treatment sessions.

As mentioned above, different experimental and partially effective methods to treat lymphedema are present and a combination of different therapeutic measures has been suggested in the literature to achieve better efficacy. Kozanoglu et al.^12^ revealed that a combination of low‐level laser and pneumatic compression treatments led to significantly better outcomes in comparison to only pneumatic compression therapy. The circumference reduction rate was 26% in that study. Lau et al.^13^ also investigated the effects of PBMT in 21 women suffering from unilateral postmastectomy lymphedema, where they found 16% reduction in the arm volume at the end of the treatment period which further increased to 28% in the follow‐up session.

We found that in patients with either “fewer removed lymph nodes” or “lesser severity of baseline lymphedema,” the response is more significant. This might be due to the existence of high interstitial pressure in cases of severe damage, which does not allow any appreciable reduction in volume and arm circumference despite the reduction in edema and stiffness. However, as we have not used skin tonometry in designing our study, we are unable to support this claim with evidence. The same problem exists in patients with a longer history of cancer treatment, who have experienced symptoms longer and did not experience favorable response to treatment for lymphedema. This might be explained by the fact that like other chronic complications, the changes tend to become less reversible as time passes after their emergence.

The discrepancies in results obtained by different studies arise from the different baseline characteristics (such as the extent of axillary dissection and underlying lymphedema severity), study designs, laser treatment duration, and laser apparatus technical specifications. More comprehensive studies are needed to reach a consensus on the efficacy of PBMT in the first place and then on the ideal settings to achieve the best outcomes.

Overall, our study had two major methodological strengths. First, we have used the water displacement method, which is a more valid method to directly determine volume changes. Second, we also used ultrasonographic evaluation to define the most probable site of fibrosis and stiffness, so our treatment was more targeted. However, it is important to mention we had some limitations in our study as well. First, we did not have a control group. We first thought that the best control method for our study would be to treat patients with a simulated laser therapy, rather than the actual laser treatment. However, this method seemed somehow nonethical to make patients already suffering from disability caused by lymphedema, to be admitted many times for such treatment. Thus, we ran our study as a single‐arm trial. Next is the lack of a comparison cohort and the small sample size does not allow to draw definitive conclusions. Moreover, a quality‐of‐life assessment was not performed. Nonetheless, we believe that this approach, using photobiomodulation, may be a precious tool in the management of this long‐term sequelae of breast cancer treatments, as it has demonstrated very promising results in our experience.

## 5 CONCLUSION

We have observed that LLLT of postmastectomy lymphedema can, at least in a complementary role and in association with current standard or classic methods in practice, be used to introduce further pain and volume reductions, thus bringing about higher patient satisfaction, encouraging patients to continue with their treatment, and raising the hope for a more active and joyful life.

## AUTHOR CONTRIBUTIONS

**Farshid Farhan**: Conceptualization; data curation. **Mahmood Samei**: Data curation; investigation. **Alireza Abdshah**: Writing—original draft; writing—review and editing. **Ali Kazemian**: Conceptualization; data curation; investigation. **Shahriar Shahriarian**: Conceptualization; data curation. **Farnaz Amouzegar‐Hashemi**: Conceptualization; supervision. **Mostafa Farzin**: Data curation; investigation. **Reza Ghalehtaki**: Formal analysis; writing—original draft. **Fatemeh Jafari**: Data curation; investigation; writing—original draft. **Francesco Cuccia**: Conceptualization; writing—original draft; writing—review and editing.

## CONFLICT OF INTEREST STATEMENT

The authors declare no conflict of interest.

## TRANSPARENCY STATEMENT

The lead author Fatemeh Jafari affirms that this manuscript is an honest, accurate, and transparent account of the study being reported; that no important aspects of the study have been omitted; and that any discrepancies from the study as planned (and, if relevant, registered) have been explained.

## Data Availability

Data can be made available upon request.
